# Significant Contribution of Evolutionary History in Coordinating Plant Size and Functional Traits in Understory Ferns of a Subtropical Secondary Forest

**DOI:** 10.3390/plants15111601

**Published:** 2026-05-23

**Authors:** Shun Zou, Chumin Huang, Xiaolong Bai, Wangjun Li, Bin He

**Affiliations:** School of Ecological Engineering, Guizhou University of Engineering Science, Bijie 551700, China; zoushun@gues.edu.cn (S.Z.); huangcm@gues.edu.cn (C.H.); baixiaolong@gues.edu.cn (X.B.); teesn470@gues.edu.cn (W.L.)

**Keywords:** plant size, functional traits, trait-size coordination, phylogeny, understory ferns, ecological strategy

## Abstract

The coordinated variation between plant size and functional traits is a critical link connecting individual ecological strategies and community assembly. However, unlike angiosperms, the drivers of trait–size coordination in coexisting fern species remain unclear. This study sampled seven coexisting fern species in a subtropical secondary forest, measuring biomass (an indicator of plant size) and functional traits related to leaf and root morphology and elemental composition. The coordinated relationship between plant individual size and functional traits was investigated using regression and principal component analysis, while the relative contributions of phylogeny, species identity, and individual biomass to trait variation were quantified via Bayesian phylogenetic generalized linear mixed models. Results indicated that there is a clear trait–size coordination relationship. Specifically, significant linear or nonlinear relationships were identified between plant size and multiple functional traits (e.g., elemental concentrations, specific leaf area, and specific root length), indicating a transition from “fast-acquisitive” to “conservative” strategies. However, variance partitioning indicated that phylogeny and species identity together explained the majority of variation in leaf and root traits (71.4% on average), whereas the independent contribution of individual biomass was minimal (7.1% on average). The results suggest that although significant trait–size coordination exists in understory fern communities, this coordination is statistically dominated by evolutionary history (phylogeny and species identity), though the ecological significance of plant size remains evident in significant trait–size coordination patterns. Overall, the coordinated variation between plant size and functional traits is pivotal in forging resource-allocation strategies and fostering fern species coexistence, highlighting that evolutionary background must be foregrounded when disentangling the mechanisms of functional community assembly.

## 1. Introduction

Plant size differences shape community functional structure and species coexistence at the interspecific scale, while intraspecific size variation reflects phenotypic plasticity enabling microhabitat adjustment [1,2,3]. As a key “hub” connecting individual performance and community dynamics, plant size provides foundational insights into coexistence mechanisms and ecosystem responses to global change.

Functional traits provide the framework for quantifying ecological strategies [4,5]. However, a key research gap remains—whether there is a universal and systematic coordinated variation between plant size and multiple functional traits. While such trait–size coordination has been extensively studied in angiosperms [6,7], it remains largely unexplored in ferns. This trait–size coordination implies that plants may form stable size–function coupling strategy combinations rather than optimizing single traits in isolation. For example, larger individuals may simultaneously have a higher specific leaf area and lower leaf dry matter concentration, indicating a fast resource acquisition strategy [8]. This coordinated structure could strengthen species coexistence by promoting niche differentiation and providing intrinsic buffering for community functional stability [9]. However, empirical evidence for trait–size coordination in natural communities is still scarce, particularly lacking systematic testing at local scales with low environmental heterogeneity and species coexistence [10,11].

Mechanistically, trait–size coordination may arise from evolutionary history (phylogenetic conservatism) or individual phenotypic plasticity [12,13,14]. Strong phylogenetic signals in functional traits have been documented in seed plants [6,15] and bryophytes [16]. Quantifying the relative contributions of evolutionary history and individual plasticity to trait–size coordination in natural communities is crucial for predicting community functional plasticity and response speed. If coordination is primarily driven by phylogeny, community structure may be stable but with limited plasticity (“evolutionary inertia”). If individual plasticity contributes significantly, the community response will be more flexible, but with potentially higher variability. Therefore, clarifying the formation mechanism requires using hierarchical models that simultaneously incorporate phylogenetic and individual variation for strict variance partitioning [17].

To systematically address this issue, this study chooses ferns as an ideal model. Ferns occupy an intermediate evolutionary position between bryophytes and seed plants, offering a unique opportunity to test whether patterns observed in angiosperms also hold in this understudied lineage. This group is characterized by strong phylogenetic conservatism, limited spore dispersal, and tightly coupled evolutionary-ecological processes [18,19], providing an excellent system for studying trait coordination in the context of evolutionary background. We conducted a whole-plant sampling of mature individuals from seven coexisting dominant fern species in a secondary forest in the Yunnan–Guizhou Plateau of China [20]. The coordinated relationship between plant individual size and functional traits was analyzed through regression and principal component analysis. Subsequently, by constructing a phylogenetic tree and applying a Bayesian phylogenetic generalized linear mixed model, we quantify the relative contributions of individual biomass, species differences, and phylogeny to the variation in leaf and root functional traits. We test two hypotheses: (H1) Plant size coordinates with functional traits in patterns consistent with the fast-slow spectrum, and (H2) this coordination is primarily structured by phylogenetic conservatism rather than individual plasticity. Answering these questions will help reveal the mechanisms behind community functional structure formation and provide important theoretical insights for understanding biodiversity maintenance and predicting community responses to global change.

## 2. Results

### 2.1. Relationship Between Plant Size and Functional Traits of Leaf and Root

**Figure 1:**
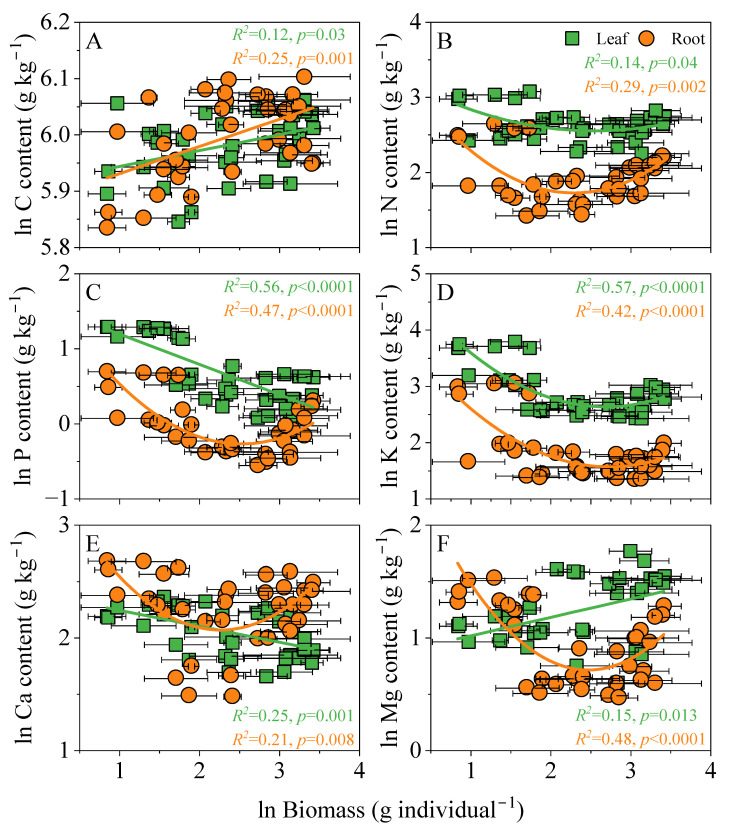
Curve fitting of leaf and root macro-element concentrations against individual size for seven dominant understory fern species in a coniferous-broadleaved mixed forest. (**A**), carbon (C) content; (**B**), nitrogen (N) content; (**C**), phosphorus (P) content; (**D**), potassium (K) content; (**E**), calcium (Ca) content; and (**F**), magnesium (Mg) content. Error bars represent standard errors (*n* = 3). The data have been log-transformed (ln).

Regression analyses indicated significant linear or quadratic relationships between individual plant size and macro-element concentrations in both leaves and roots (Figure 1). In leaves, C (*R*^2^ = 0.12, *p* = 0.030) and Mg (*R*^2^ = 0.15, *p* = 0.013) contents increased significantly with biomass, whereas P (*R*^2^ = 0.56, *p* < 0.0001) and Ca (*R*^2^ = 0.25, *p* = 0.001) contents declined markedly with increasing biomass. N (*R*^2^ = 0.14, *p* = 0.040) and K (*R*^2^ = 0.57, *p* < 0.0001) contents showed U-shaped responses, with minima at 12.91 g kg^−1^ (natural logarithm value = 2.56) and 14.85 g kg^−1^ (natural logarithm value = 2.70), respectively. In roots, only C concentration increased linearly with biomass (*R*^2^ = 0.25, *p* = 0.001). N, P, K, Ca, and Mg concentrations all exhibited significant U-shaped curves, with turning points at 9.99, 12.51, 15.52, 9.62, and 11.91 g kg^−1^ (natural logarithm value 2.30–2.74), respectively.

**Figure 2:**
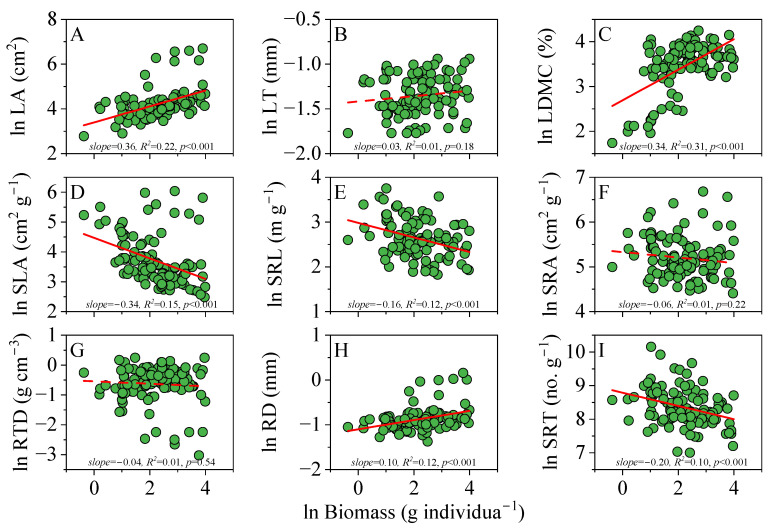
Linear regression of leaf and root morphological traits against individual size for seven dominant understory fern species in a coniferous-broadleaved mixed forest. (**A**), leaf area (LA); (**B**), leaf thickness (LT); (**C**), leaf dry mass content (LDMC); (**D**), specific leaf area (SLA); (**E**), specific root length (SRL); (**F**), specific root surface area (SRA); (**G**), root tissue density (RTD); (**H**), average root diameter (RD); and (**I**), specific root tip number (SRT). The data have been log-transformed (ln). A *p*-value < 0.05 gives a solid red regression line; otherwise, a red dashed line.

Linear regression analyses of leaf and root morphological traits against individual size indicated significant relationships (Figure 2). For leaf traits, LA (*R*^2^ = 0.22, *p* < 0.001) and LDMC (*R*^2^ = 0.31, *p* < 0.001) increased significantly with individual biomass, while SLA (*R*^2^ = 0.15, *p* < 0.001) decreased significantly as individual biomass increased. In contrast, LT showed no significant relationship with individual biomass (*p* = 0.18). For root traits, RD (*R*^2^ = 0.12, *p* < 0.001) increased significantly with individual biomass, whereas SRL (*R*^2^ = 0.12, *p* < 0.001) and SRT (*R*^2^ = 0.10, *p* < 0.001) both decreased significantly. However, SRA (*p* = 0.22) and RTD (*p* = 0.54) showed no significant relationship with individual biomass.

**Figure 3:**
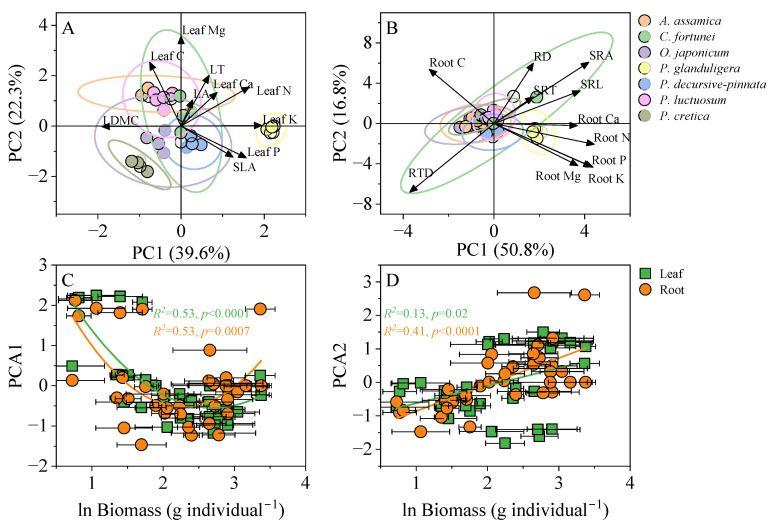
Principal component analysis of leaf (**A**) and root (**B**) functional traits and curve fitting of the first two principal component axes ((**C**), PCA1; (**D**), PCA2) against plant individual size, for seven dominant understory fern species in a coniferous-broadleaved mixed forest. C, carbon content; N, nitrogen content; P, phosphorus content; K, potassium content; Ca, calcium content; Mg, magnesium content; LA, leaf area; LT, leaf thickness; LDMC, leaf dry mass content; SLA, specific leaf area; SRL, specific root length; SRA, specific root surface area; RTD, root tissue density; RD, average root diameter; and SRT, specific root tip number. Error bars represent standard errors (n = 3).

Further principal component analyses (PCA) were conducted separately for leaf and root functional traits, and the first two principal components (PCA1 and PCA2) were extracted for each organ. For leaves, PCA1 explained 39.6% of the total trait variation, primarily associated with leaf K, LDMC, P, N, and SLA, while PCA2 explained 22.3% of the variation, mainly driven by leaf Mg, C, LT, LA, and Ca (Figure 3A). Leaf PCA1 showed a significant quadratic relationship with individual biomass (*R*^2^ = 0.53, *p* < 0.0001), first decreasing and then increasing, with a turning point at 14.81 g kg^−1^ (natural logarithm value = 2.70). In contrast, PCA2 exhibited a significant positive linear relationship with individual biomass (*R*^2^ = 0.13, *p* = 0.02; Figure 3C). For roots, PCA1 accounted for 50.8% of the total trait variation, with major contributions from root Ca, N, SRL, P, K, SRA, and RTD, whereas PCA2 explained 16.8% of the variation, mainly influenced by root RD, SRT, and C (Figure 3B). Similarly, root PCA1 displayed a significant quadratic relationship with individual biomass (*R*^2^ = 0.53, *p* = 0.0007), first decreasing and then increasing, with a turning point at 9.70 g kg^−1^ (natural logarithm value = 2.27). PCA2 of roots increased significantly with individual biomass in a linear manner (*R*^2^ = 0.41, *p* < 0.0001; Figure 3D).

### 2.2. Phylogenetic Signal of Traits

Phylogenetic signal analysis for the seven dominant understory fern species is summarized in Table 1. Biomass showed *K* = 1.12 (*p_K* = 0.10) and *λ* = 1.00 (*p_λ* = 0.16), with non-significant *p*-values precluding strong evolutionary inferences. Among leaf elemental traits, leaf Mg exhibited the highest *K* (1.54, *p_K* < 0.01) but marginal *p_λ* (0.05); leaf P showed *K* = 1.22 (*p_K* = 0.06) with non-significant *p_λ* (0.11); leaf K had *K* = 1.00 (*p_K* = 0.04) and *p_λ* = 0.29. For all three, the non-significant *p_λ* values indicate that the null hypothesis of no phylogenetic signal cannot be rejected. Leaf C, N, and Ca showed weaker or non-significant signals. For leaf structural traits, LDMC was the only trait with a significant *K* (0.88, *p_K* = 0.02), though *λ* (0.77, *p_λ* = 0.53) was non-significant. LA, LT, and SLA exhibited low and non-significant signals (*λ* = 0.00, *p_λ* = 1.00 for all). Root traits predominantly showed *K* < 1 with non-significant *λ* (all *p_λ* ≥ 0.57, most = 1.00), suggesting limited phylogenetic structuring in belowground traits. Root K was the only root trait with a significant *K* (0.78, *p_K* < 0.01), but its *λ* was non-significant (0.35, *p_λ* = 0.81). For PCA axes, leaf PCA2 showed *K* = 1.37 (*p_K* = 0.01; *λ* = 1.00, *p_λ* = 0.08); leaf PCA1 had *K* = 0.80 (*p_K* = 0.04; *λ* = 0.46, *p_λ* = 0.81); root PCA components were non-significant.

Given the limited species pool (*n* = 7), statistical power to detect significant phylogenetic signals is low. Therefore, non-significant *p_λ* results (*p* > 0.05) should be interpreted with caution, and we avoid overinterpreting *K* values without statistical support.

### 2.3. Effects of Biomass, Phylogeny, and Species on Leaf and Root Functional Traits

**Figure 4:**
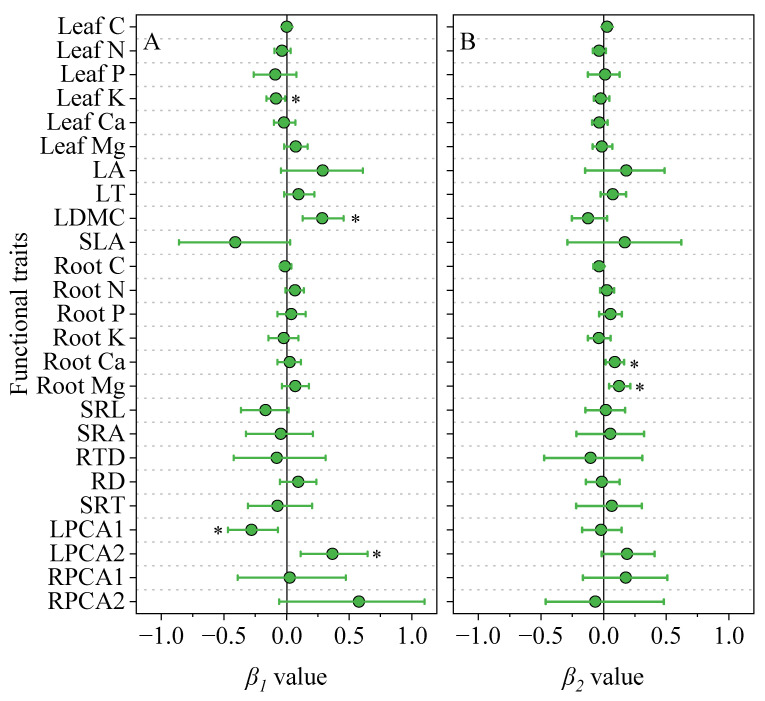
Fitted first-order ((**A**), *β*_1_) and second-order ((**B**), *β*_2_) coefficient means and 95% credible intervals for leaf and root functional traits and their first two principal axes of seven dominant understory fern species in a coniferous-broadleaved mixed forest, based on a Bayesian phylogenetic linear mixed model. * Indicates that the 95% credible interval does not overlap 0. Fixed effect: individual plant size (biomass); random effects: phylogeny and species. C, carbon content; N, nitrogen content; P, phosphorus content; K, potassium content; Ca, calcium content; Mg, magnesium content; LA, leaf area; LT, leaf thickness; LDMC, leaf dry mass content; SLA, specific leaf area; SRL, specific root length; SRA, specific root surface area; RTD, root tissue density; RD, average root diameter; and SRT, specific root tip number. LPCA1, PCA1 of leaf functional traits; LPCA2, PCA2 of leaf functional traits; RPCA1, PCA1 of root functional traits; RPCA2, PCA2 of root functional traits.

The PGLMM model was used to assess the influence of plant size (biomass) as a fixed effect on leaf and root traits and their principal component axes (PCA1 and PCA2). After setting phylogeny and species as random effects, only a small subset of functional traits showed significant linear or quadratic relationships with biomass (Figure 4). Among these, the linear coefficients β_1_ for leaf K (−0.16 to −0.01), LDMC (0.13 to 0.46), LPCA1 (−0.47 to −0.07), and LPCA2 (0.11 to 0.65) were significant (95% credible intervals excluded 0), as were the quadratic coefficients β_2_ for root Ca (0.02 to 0.16) and Mg (0.04 to 0.21).

**Figure 5:**
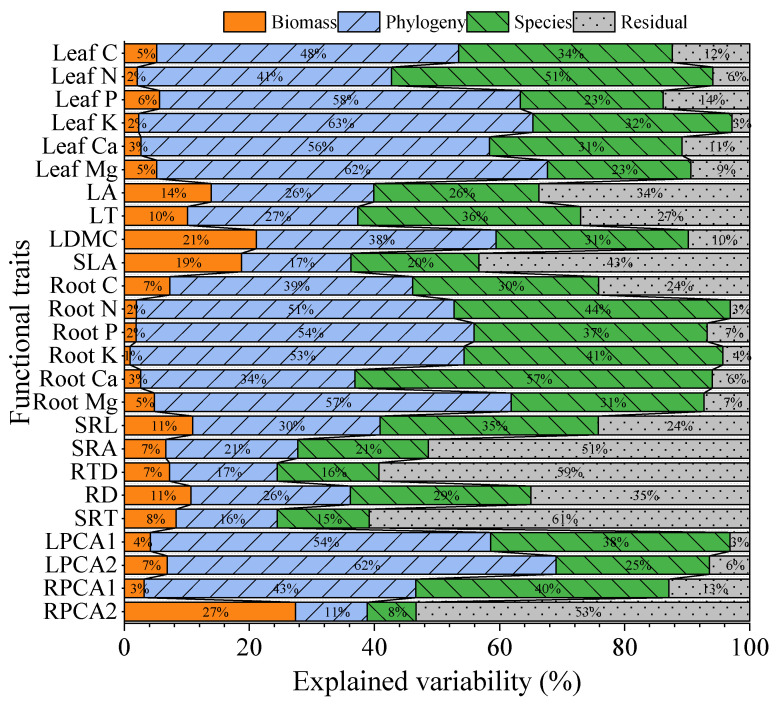
Proportion of explained variability in leaf and root functional traits and their first two principal axes explained by fixed and random effects in a Bayesian phylogenetic linear mixed model for seven dominant understory fern species in a coniferous-broadleaved mixed forest. Fixed effect: individual plant size (biomass); random effects: phylogeny and species; Residual: unexplained portion. C, carbon content; N, nitrogen content; P, phosphorus content; K, potassium content; Ca, calcium content; Mg, magnesium content; LA, leaf area; LT, leaf thickness; LDMC, leaf dry mass content; SLA, specific leaf area; SRL, specific root length; SRA, specific root surface area; RTD, root tissue density; RD, average root diameter; and SRT, specific root tip number. LPCA1, PCA1 of leaf functional traits; LPCA2, PCA2 of leaf functional traits; RPCA1, PCA1 of root functional traits; RPCA2, PCA2 of root functional traits.

For leaf functional traits, phylogeny explained on average 43.7% (17.5–63.0%) of the variance, and species accounted for 30.7% (20.4–51.4%), whereas biomass contributed only 8.6% (2.1–21.1%). For root traits, phylogeny averaged 36.2% (16.2–57.0%), species 32.4% (14.7–57.2%), and biomass merely 5.7% (0.9–10.9%). Overall, phylogeny explained 54.4% and 62.2% of the variance in leaf PCA1 and PCA2, respectively, species 38.3% and 24.6%, and biomass 4.1% and 6.8%. For root PCA axes, phylogeny accounted for 43.4% and 11.4%, species for 40.5% and 7.8%, and biomass for 3.2% and 27.4%, respectively. Variance partitioning analysis indicated that phylogeny and species together explained most of the variation in traits (Figure 5).

## 3. Discussion

### 3.1. Coordinated Variation Between Plant Individual Size and Functional Traits

Our results support H1: plant size coordinates with functional traits in patterns consistent with strategy transitions. Leaf C and Mg increased with biomass, while P and Ca decreased (Figure 1), suggesting shifts from rapid resource acquisition toward structural reinforcement and conservative use [4,8]—consistent with the fast-slow spectrum but expressed within phylogenetically constrained bounds. This pattern aligns with observations in angiosperms [6,7], where larger individuals also shift toward conservative strategies, suggesting a general principle of plant size–function coupling across vascular plants. The decrease in P concentration suggests that larger individuals are more likely to allocate resources toward structural reinforcement and stress resistance [21]. Larger individuals adopted conservative strategies—reduced tissue nutrients and extended organ lifespan—enhancing nutrient use efficiency and stabilizing resource utilization [22], consistent with our H2 that evolutionary history constrains the expression of size-dependent plasticity.

The root results further support this idea, as C concentration in roots increased linearly with biomass, while N, P, K, Ca, and Mg concentrations followed U-shaped curves, with turning points at 9.99, 12.51, 15.52, 9.62, and 11.91 g kg^−1^, respectively (Figure 2). These results further confirm the differences in leaf and root resource allocation strategies among mature fern individuals from different species within the same community. Similar trends have been validated in several ecological studies. For instance, Jiang et al. (2024) [23] found that, in coastal plant communities, root resource allocation exhibited similar nonlinear changes as individual biomass increased, supporting the relationship between plant size and resource acquisition strategies. The observed turning points may correspond to key life-history transitions, such as the onset of spore production in ferns, when resources shift from vegetative growth to reproduction. Direct validation is not possible due to lack of reproductive status data, but future studies incorporating life-stage information could clarify this link. In terms of root morphological traits, RD significantly increased with individual biomass, while SRL and SRT significantly decreased (Figure 3). These changes reflect a shift in root growth strategy from rapid exploration to a more structured and durable root system as individuals increase in size. Similar findings have been reported by Weigelt et al. (2021) [22], who observed that as plant size increases, root exploration traits gradually shift toward more stable structural forms, enhancing resource acquisition stability. SRA and RTD did not show significant relationships with individual biomass, which may be attributed to differences in root strategies across different habitats (Figure 3). This result is similar to findings by Jiang et al. (2024) [23] in coastal plants. Principal component analysis further indicated significant nonlinear relationships between leaf and root traits and plant size. The first principal component of leaf and root traits explained 39.6% and 50.8% of trait variation, respectively, and showed significant quadratic relationships with individual biomass (Figure 3). The observed U-shaped and quadratic relationships between biomass and nutrient concentrations may reflect: (i) ontogenetic shifts in resource allocation priorities [21]; (ii) size-dependent changes in metabolic scaling [5,9]; (iii) species turnover along the size gradient; or (iv) nonlinear plastic responses to microenvironmental heterogeneity. We cannot distinguish these mechanisms with the current design; thus, we interpret these patterns as consistent with, but not definitive evidence for, size-dependent strategy transitions. The “turning points” identified statistically (e.g., ln-biomass 2.70 for leaf PCA1) should be interpreted as descriptive inflection points rather than adaptive optima, pending experimental validation [22]. When comparing our findings with global trait models, we found that the relationship between size and functional traits holds both within and between species. Consistent with Wright et al. (2004), we found that SLA decreases with plant size, while LDMC increases, a pattern that also holds in mixed-species samples [1,8]. A key finding is the shift of trait allocation toward the conservative end as individuals grow, challenging the linear assumption of size-trait relationships in environmental context [24,25,26]. Our data supports the view that trait models must account for nonlinearity, as ecological processes differ significantly before and after the optimal biomass threshold [14]. This nonlinear size-function relationship suggests that functional diversity arises not only from species turnover but also from individual phenotypic rearrangement along the size axis [27].

In conclusion, our findings suggest that plant size not only influences leaf and root element allocation but also drives the transition from “fast-acquisitive” to “conservative/structural” strategies by modulating chemical composition and morphological traits. This transition not only helps plants optimize their growth strategies in resource-limited environments but also provides a mechanistic basis for species coexistence. The coordinated variation between individual size and functional traits plays a significant role in the coexistence of understory fern species. In forest understories, individuals with different sizes and functional traits can occupy distinct ecological niches through different resource allocation and growth strategies, thereby promoting species coexistence [3,28,29,30,31]. While our findings align with patterns documented in angiosperms and bryophytes [6,7,16], ferns occupy an intermediate evolutionary position, and their trait–size coordination shows both similarities to and differences from these lineages. Future comparative studies across vascular plants are needed to assess the generality of these patterns. While this study provides insights into trait–size coordination in a relatively homogeneous secondary forest, we acknowledge that such relationships may vary across forest successional stages. For example, ferns in pioneer forests typically exhibit shorter leaf lifespans and faster resource turnover, which may lead to stronger acquisitive strategies and potentially different trait–size coordination patterns. Therefore, future studies should explicitly compare multiple successional stages to evaluate the generality of our findings.

### 3.2. Size–Trait Coordination Dominated by Evolutionary History

**Figure 6:**
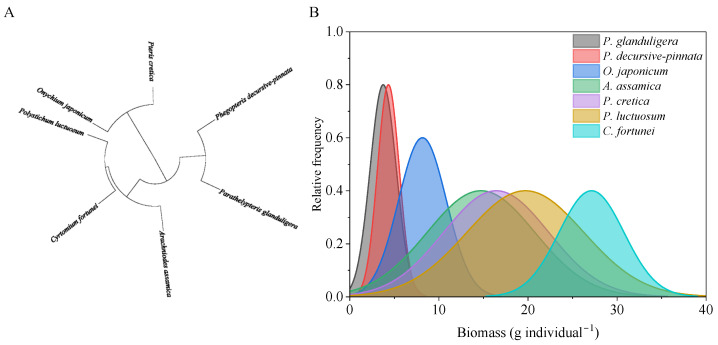
Phylogenetic tree (**A**) and individual size (**B**) of seven dominant fern species in the understory of a mixed coniferous-broadleaved forest.

This study investigates the coordinated variation between plant size (biomass) and functional traits, highlighting the greater contribution of evolutionary history—specifically phylogeny and species identity—in mediating trait–size relationships. PGLMM models indicated that phylogeny accounted for 43.7% and 36.2% of the variance in leaf and root traits, respectively, whereas biomass contributed only 8.6% and 5.7% (Figure 6). These results suggest that trait–size coordination is not merely a function of individual size but is profoundly dominated by species’ phylogenetic background, reinforcing the importance of evolutionary history in trait expression [32,33]. This strong phylogenetic constraint is consistent with large-scale patterns observed in angiosperms [6,15] and bryophytes [16], suggesting that evolutionary history plays a foundational role in structuring functional trait variation across divergent land plant lineages. In particular, leaf and root elemental composition (e.g., C, N, Mg, and P) exhibited strong phylogenetic conservatism, indicating that closely related species share similar functional trait values [34,35,36]. These findings demonstrate that evolutionary history exerts a stronger influence on trait–size relationships than does individual size per se [37,38].

Notably, leaf elemental composition was strongly influenced by genetic background, underscoring the phylogenetic constraints on elemental allocation. Although phylogenetic effects were slightly weaker in roots, they remained statistically significant [39]. While plant size exerted some influence on traits, its independent contribution was marginal after accounting for species and phylogenetic effects. PGLMM results confirmed that the explanatory power of biomass diminished substantially once evolutionary constraints were considered, supporting the view that interspecific and phylogenetic variations outweigh intraspecific size effects in shaping trait patterns. Thus, functional trait expressions in plant communities appear to be strongly influenced by deep evolutionary legacies rather than individual-level plasticity [35,36]. Despite the strong phylogenetic signal, we also observed limited flexibility in how plant size modulates resource acquisition strategies and functional traits. Smaller plants tended toward rapid growth and resource-acquisitive traits, whereas larger plants invested more in structural integrity and stress tolerance. Nevertheless, this plasticity remained bounded within phylogenetically defined constraints. As noted by Cadotte et al. (2013) [38], while ecological factors fine-tune trait expression, the scope of such adjustments is ultimately dominated by species identity and evolutionary history. In essence, evolutionary history defines the “trait–size possibility space,” within which individual plants adjust their functional traits in response to size-dependent and environmental conditions [3,30,40]. We emphasize that variance partitioning quantifies the hierarchical sources of trait variation but does not imply that lower-variance components lack ecological importance. The modest independent contribution of biomass (7.1% on average) reflects its statistical effect after accounting for phylogenetic and species-level covariance, not the adaptive irrelevance of size-mediated plasticity. Indeed, the significant trait–size correlations observed (Figure 1, Figure 2 and Figure 3) suggest that plant size remains an ecologically meaningful axis of functional variation, even when evolutionary history accounts for most among-species differences. The pooling procedure for elemental analyses constrains our ability to detect individual-level plasticity. By averaging across three individuals, we effectively reduce the within-species variance component, which may inflate the relative importance of phylogeny and species identity in variance partitioning. Consequently, our estimates of biomass effects (7.1% average) should be considered conservative lower bounds, and the true contribution of phenotypic plasticity may be higher.

Among understory ferns, the coordination between plant size and functional traits facilitates adaptation to heterogeneous resource environments. Smaller individuals often adopted a “fast-acquisitive” strategy, emphasizing efficient resource uptake and utilization, while larger individuals favored a “structural conservation” strategy, enhancing stability and endurance [5,41]. This divergence in resource allocation promotes niche differentiation, reduces interspecific competition, and ultimately fosters species coexistence [42,43,44]. Evolutionary history plays a foundational role in this process by shaping the genetic and trait-based toolkit available to species, thereby conditioning their adaptive responses. For instance, larger fern lineages may evolve traits suited to low-light and nutrient-poor soils, whereas smaller lineages prioritize high photosynthetic rates and rapid growth [18,29,45,46]. These phylogenetically structured trait differences provide a mechanistic basis for niche partitioning and enhance biodiversity within plant communities [14,38]. Notably, our findings should be interpreted as context-dependent rather than universal patterns. Caution is warranted in extending these findings to other plant groups or environmental contexts. The strong phylogenetic effects observed here may reflect the particular evolutionary history of fern lineages with limited dispersal and conserved niche preferences [18,19]. The strong phylogenetic effects observed here may partly reflect the limited environmental variation captured in our single-plot sampling design. In more heterogeneous environments, individual plasticity and microenvironmental filtering might play larger roles. Therefore, our findings should be interpreted as context-dependent, and future studies should explicitly sample across multiple microhabitats and environmental gradients to assess the generality of phylogenetic dominance in trait–size coordination. Additionally, our single-time sampling, conducted during the peak growing season, cannot capture seasonal trait dynamics (e.g., N, SLA, and SRL). Whether the observed patterns represent stable ecological strategies or a phenological snapshot remains unclear and should be addressed by future multi-season studies.

## 4. Materials and Methods

### 4.1. Study Site

The study was conducted in Nanshan Park (105°18′2.95″ E, 27°16′22.55″ N), located in Bijie City, Guizhou Province, southwestern China, on the northeastern margin of the Yunnan–Guizhou Plateau. The area is characterized by mid-mountain landforms with an elevation of approximately 1500 m a.s.l. Influenced by the subtropical monsoon, the regional climate is cool and humid. Mean annual air temperature is 13.5 °C (January 3.1 °C and July 22.2 °C), mean annual relative humidity is 80.3%, and mean annual precipitation is 886 mm, of which more than 70% falls between May and September. The growing season extends from mid-April to late October.

The dominant vegetation in Nanshan Park is a secondary successional deciduous broadleaved forest more than 60 years old, with a canopy cover of approximately 70%. Beneath this open canopy, light availability is high, and understory species richness is substantial. Canopy dominants include *Populus adenopoda* Maxim., *Betula luminifera* H. J. P. Winkl., and *Quercus aliena* Blume. The shrub layer is co-dominated by *Cotoneaster franchetii* Bois, *Hypericum monogynum* L., and *Viburnum foetidum var. ceanothoides* (C. H. Wright) Hand.-Mazz., and the herb layer is characterised by pteridophytes such as *Pteris cretica* L. and graminoids such as *Arthraxon prionodes* (Steud.) Dandy.

### 4.2. Field Sampling

During the middle of growing season of 2024 (July–August), when leaves were fully expanded, we established a 1 ha plot (100 m × 100 m) within the deciduous broadleaved mixed forest of Nanshan Park. All dominant fern individuals (>15 plants per species) were harvested whole. Given fern root systems are typically compact and shallow (<15 cm depth for the study species), plants were excavated with a spade to 20 cm depth and 15 cm radius around the rhizome and then gently lifted and transported intact to the laboratory. In the lab, root systems were washed over a 0.5 mm sieve to recover fine roots. In total, seven fern species belonging to three families and seven genera were collected, including *Parathelypteris glanduligera* (Kunze) Ching, *Phegopteris decursive-pinnata* (H. C. Hall) Fée, *Onychium japonicum* (Thunb.) Kunze, *Arachniodes assamica* (Kuhn) Ohwi, *Pteris cretica* L., *Polystichum luctuosum* (Kunze) T. Moore (recorded as *Polystichum tsus-simense* (Hook.) J. Sm. in Flora of China), and *Cyrtomium fortunei* J. Sm. These species exhibit substantial variation in average individual size, spanning a relatively broad gradient of individual biomass (0.70–54.25 g individual^−1^, Figure 6). For each species, 15 healthy, intact plants without visible disease, pest, or herbivore damage were selected, yielding 105 fern individuals. All samples were sealed in plastic bags and transported to the laboratory for subsequent analyses.

### 4.3. Measurement of Biomass and Morphology Traits of Leaf and Root

After transport to the laboratory, each plant was separated into organs (leaf, stem, and root) and placed in individual sealed bags. Stems were rinsed to remove soil and oven-dried at 70°C for 48 h (0.0001 g precision) to obtain stem biomass (S-Biomass, g). From each leaf sample, 3–5 leaves were selected to determine morphological traits: leaf area (LA, cm^2^), leaf thickness (LT, mm), leaf dry matter content (LDMC, %), and specific leaf area (SLA, cm^2^ g^−1^). The remaining leaves were oven-dried to yield partial leaf biomass (L1-Biomass, g). LA was calculated as total scanned leaf area divided by leaf number using an HP Scanjet M231 imaging system. LT was measured as the mean of three readings taken with a micrometer (0.001 mm precision) away from the main vein. After these measurements, fresh mass was recorded, leaves were oven-dried, and dry mass (L2-Biomass, g) was obtained. LDMC was expressed as dry mass per unit fresh mass, and SLA as total leaf area divided by dry mass [47].

Roots were gently washed, and an intact subsample (selected following standardized criteria: (i) representative of the full root system in diameter distribution (visually confirmed to include fine, medium, and coarse roots); (ii) free of obvious damage, disease, or herbivory; and (iii) approximately 20–30% of total fresh root mass) was used to determine morphological indices: specific root length (SRL, m g^−1^), specific root surface area (SRA, cm^2^ g^−1^), root tissue density (RTD, g cm^3^), mean root diameter (RD, mm), and specific root tip number (SRT, no. g^−1^). The remaining roots were oven-dried to yield root biomass (R1-Biomass, g). Root length, surface area, volume, RD, and tip number were quantified with a flatbed scanner coupled to DJ-GX02 imaging software (Dianjiang Tech, Shanghai, China), after which samples were oven-dried and weighed (R2-Biomass, g). SRL, SRA, and SRT were calculated by dividing root length, surface area, and tip number by root dry mass, respectively; RTD was obtained by dividing root dry mass by root volume [47].

Total plant biomass of individual was calculated as the sum of S-Biomass, L1-Biomass, L2-Biomass, R1-Biomass, and R2-Biomass and used as an indicator of plant size (Biomass, g individual^−1^).

### 4.4. Measurement of Macro-Elements in Leaf and Root

Due to limited tissue mass for elemental analysis (a minimum of ca. 200 mg dried material per sample to accommodate both CN combustion and ICP-AES multi-element determination after acid digestion), leaves and roots from three similarly sized individuals per species were pooled into composite samples (n = 35 total). This pooling reduces estimates of intraspecific variability and may underestimate the contribution of individual biomass to trait variation, representing a conservative bias toward phylogenetic and species effects. Leaf and root C (g kg^−1^) and N (g kg^−1^) contents were quantified by high-temperature combustion on a Vario MAX CN analyzer (Elementar, Hanau, Germany). P (g kg^−1^), K (g kg^−1^), Ca (g kg^−1^), and Mg (g kg^−1^) contents were determined after acid digestion (HNO_3_/HClO_4_, 4:1, *v*/*v*) with an iCAP 7400 ICP-AES (Thermo Fisher, Bremen, Germany).

### 4.5. Statistical Analysis

First, species’ Latin names were standardized against The Plant List (http://www.theplantlist.org (accessed on 20 December 2025)). The phylogeny of the seven fern species was then constructed using the V.PhyloMaker2 package in R version 4.4.3 (R Core Team 2025, Figure 1). To examine relationships between plant size (biomass) and functional traits, we fitted linear and quadratic models to leaf and root traits (element concentrations and morphological characters) against biomass in OriginPro 2024b (OriginLab Corporation, Northampton, MA, USA) and generated corresponding plots. Principal component analyses were performed separately for leaf and root traits to extract the first two axes (LPCA1, LPCA2, RPCA1, and RPCA2), which were likewise regressed against biomass using linear or quadratic functions to assess coordinated changes. Data were log-transformed prior to analysis. Both linear and quadratic models were fitted to capture potential nonlinear size–trait relationships predicted by metabolic scaling theory and optimal partitioning models. Quadratic terms allow detection of U-shaped or hump-shaped patterns indicative of size-dependent strategy transitions. Model selection was based on deviance information criterion (DIC); quadratic terms were retained when 95% credible intervals for the second-order coefficient excluded 0.

Phylogenetic signals of each trait and principal component axis were estimated in R with the phylosig function (picante package, 999 randomizations). Blomberg’*K* and Pagel’*λ* values were calculated to evaluate trait distribution on phylogeny. *K* compares observed trait clustering to a random model (*K* > 1 indicated clustered, *K* < 1 indicated overdispersed, and *K* = 1 indicated random). *λ* measures phylogenetic correlation (*λ* ≈ 1 indicated strong phylogenetic signal, and *λ* ≈ 0 indicated no correlation) [48].

To test whether coordinated variation between plant size and functional traits is shaped by evolutionary processes, we employed a phylogenetic generalized linear mixed model (PGLMM) using the *MCMCglmm* package [49]. Continuous traits were fitted with a Gaussian family. Biomass effects were modelled with both linear and quadratic terms to capture potential nonlinearity. The phylogeny (ape package) was converted to a variance-covariance matrix and included to control for non-independence among species. Random effects comprised species and phylogeny, accounting for interspecific and phylogenetic correlations. Weakly informative priors ensured MCMC stability. Chains ran for 52,000 iterations, with 2000 burn-in and thinning every 50 iterations to ensure convergence. Posterior means and 95% credible intervals of linear and quadratic biomass coefficients were extracted. Variance partitioning quantified the relative contributions of biomass, phylogeny, species, and residual error to trait variation. Visualizations were completed in OriginPro 2024b.

## 5. Conclusions

This study supports that in understory fern communities, plant size and functional traits covary systematically. Smaller individuals pursue rapid resource acquisition strategies, while larger individuals shift toward structural reinforcement and conservative resource use. This size-based trait coordination may contribute to niche differentiation in the forest understory and promote species coexistence. However, our conclusion that evolutionary history dominates trait variation should be interpreted with caution, as the pooling of samples may have underestimated individual-level variation. Future studies with individual-level elemental measurements are needed to validate these findings.

## Figures and Tables

**Table 1:** Phylogenetic signal of functional traits and principal component axes of seven dominant understory fern species in a coniferous-broadleaved mixed forest.

Variables *	*K*	*p*_K	*λ*	*p*_*λ*
Biomass	1.12	0.10	1.00	0.16
Leaf C	0.88	0.13	0.59	0.48
Leaf N	0.50	0.59	0.00	1.00
Leaf P	1.22	0.06	1.00	0.11
Leaf K	1.00	0.04	1.00	0.29
Leaf Ca	0.85	0.14	0.55	0.50
Leaf Mg	1.54	0.00	1.00	0.05
LA	0.55	0.48	0.00	1.00
LT	0.43	0.79	0.00	1.00
LDMC	0.88	0.02	0.77	0.53
SLA	0.53	0.43	0.00	1.00
Root C	0.58	0.38	0.00	1.00
Root N	0.69	0.20	0.20	0.77
Root P	0.71	0.22	0.00	1.00
Root K	0.78	0.00	0.35	0.81
Root Ca	0.44	0.81	0.00	1.00
Root Mg	0.80	0.16	0.00	1.00
SRL	0.49	0.56	0.00	1.00
SRA	0.48	0.57	0.00	1.00
RTD	0.45	0.67	0.00	1.00
RD	0.42	0.90	0.00	1.00
SRT	0.44	0.73	0.00	1.00
LPCA1	0.80	0.04	0.46	0.81
LPCA2	**1.37**	**0.01**	1.00	0.08
RPCA1	0.65	0.24	0.00	1.00
RPCA2	0.88	0.12	0.91	0.57

* Note: C, carbon content; N, nitrogen content; P, phosphorus content; K, potassium content; Ca, calcium content; Mg, magnesium content; LA, leaf area; LT, leaf thickness; LDMC, leaf dry mass content; SLA, specific leaf area; SRL, specific root length; SRA, specific root surface area; RTD, root tissue density; RD, average root diameter; SRT, specific root tip number. LPCA1, PCA1 of leaf functional traits; LPCA2, PCA2 of leaf functional traits; RPCA1, PCA1 of root functional traits; RPCA2, PCA2 of root functional traits.

## Data Availability

The data files are shared via the following dataset: zou, shun (2026), “Dominant Role of Evolutionary History in Coordinating Plant Size and Functional Traits in Understory Ferns of a Subtropical Secondary Forest,” Mendeley Data, V1, https://doi.org/10.17632/h3c3r7v45c.1.

## Abbreviations

The following abbreviations are used in this manuscript:

C	carbon content
N	nitrogen content
P	phosphorus content
K	potassium content
Ca	calcium content
Mg	magnesium content
LA	leaf area
LT	leaf thickness
LDMC	leaf dry mass content
SLA	specific leaf area
SRL	specific root length
SRA	specific root surface area
RTD	root tissue density
RD	average root diameter
SRT	specific root tip number
LPCA1	PCA1 of leaf functional traits
LPCA2	PCA2 of leaf functional traits
RPCA1	PCA1 of root functional traits
RPCA2	PCA2 of root functional traits
